# Supplementary material for: Rhizoboxes as Rapid Tools for the Study of Root Systems of Prunus Seedlings
Source: Plants (Basel). 2022 Aug 9;11(16):2081. doi: 10.3390/plants11162081 (PMC9416644; doi:10.3390/plants11162081)
Supplement: Supplementary file 1 [file plants-11-02081-s001.zip › plants-1820545-supplementary.pdf]

Technical note

Rhizoboxes as Rapid Tools for the Study of Root Systems of Prunus Seedlings

Ricardo A. Lesmes-Vesga[1], Liliana M. Cano[2], Mark A. Ritenour[1], Ali Sarkhosh[3], José X. Chaparro[3] and Lorenzo Rossi[1,*]

[1] Horticultural Sciences Department, Indian River Research and Education Center, Institute of Food and Agricultural Sciences, University of Florida, Fort Pierce, FL 34945, USA

[2] Plant Pathology Department, Indian River Research and Education Center, Institute of Food and Agri-cultural Sciences, University of Florida, Fort Pierce, FL 34945, USA

[3] Horticultural Sciences Department, Institute of Food and Agricultural Sciences, University of Florida, Gainesville, FL 32603, USA

*For correspondence: l.rossi@ufl.edu; Tel.: +1-772-577-7341

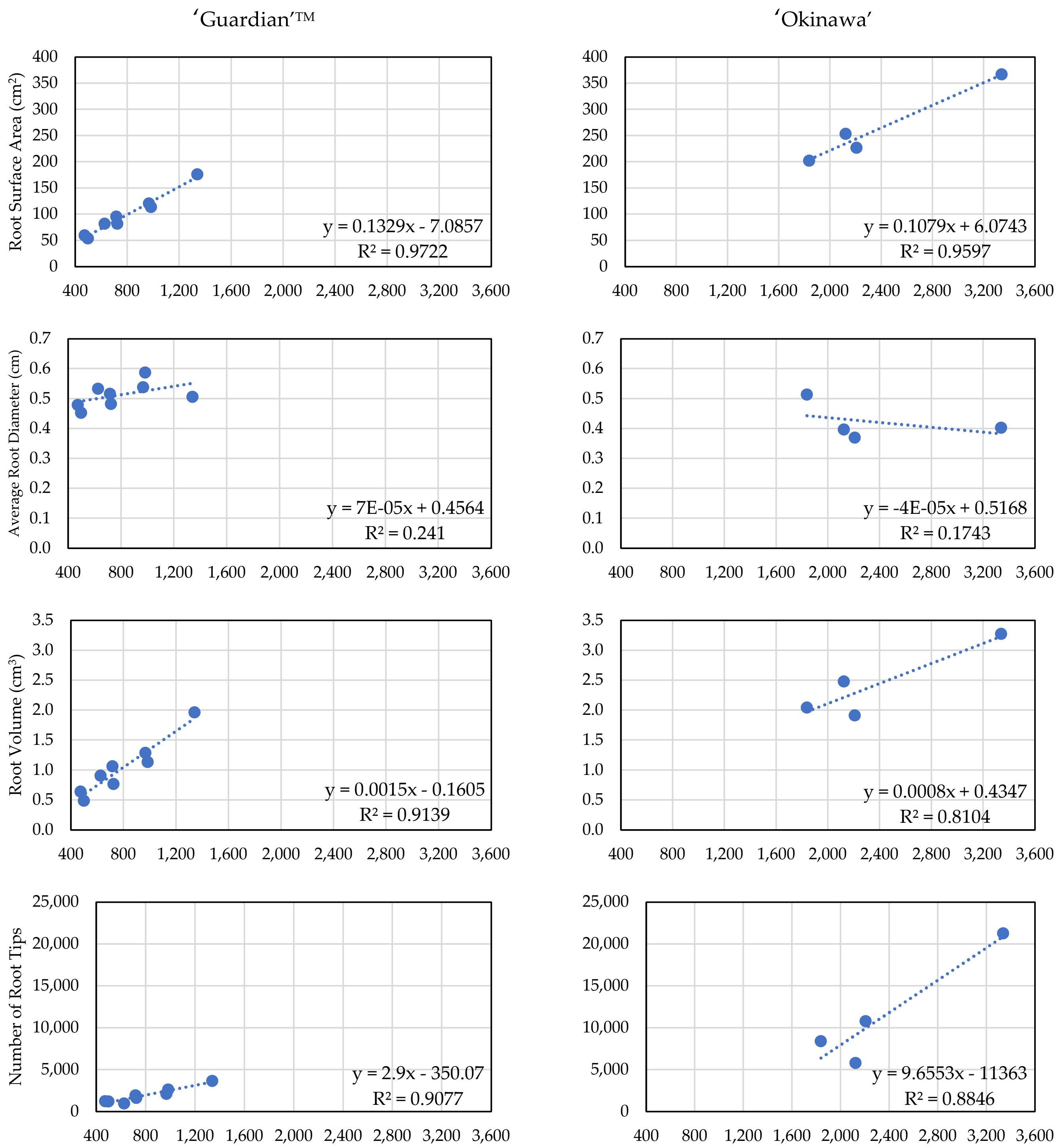
'Guardian'™
'Okinawa'
Root Surface Area (cm²)
y = 0.1329x - 7.0857
R² = 0.9722
y = 0.1079x + 6.0743
R² = 0.9597
Average Root Diameter (cm)
y = 7E-05x + 0.4564
R² = 0.241
y = -4E-05x + 0.5168
R² = 0.1743
Root Volume (cm³)
y = 0.0015x - 0.1605
R² = 0.9139
y = 0.0008x + 0.4347
R² = 0.8104
Number of Root Tips
y = 2.9x - 350.07
R² = 0.9077
y = 9.6553x - 11363
R² = 0.8846

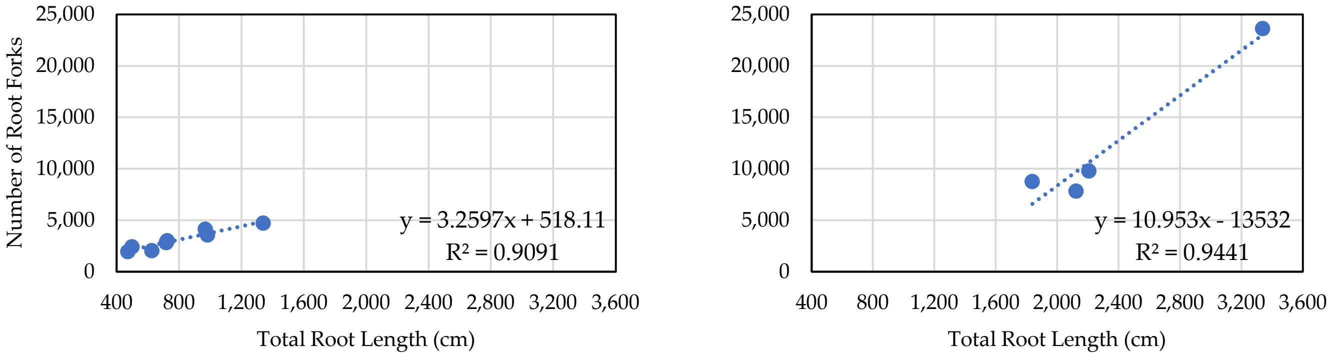

Figure S1. Linear regressions of root morphological parameters in 'Okinawa' and 'Guardian'TM rootstocks for total root length, total root surface area, average root diameter, total root volume, number of root tips, and number of root forks.

Table S1: Linear regressions R^2 values of root morphological parameters in 'Okinawa' and 'Guardian'™ rootstocks for total root length, total root surface area, average root diameter, total root volume, number of root tips, and number of root forks.

	**Total Root Length – Linear regressions (R^2)**	
	**'Guardian'™**	**'Okinawa'**
Root Surface Area (cm^2)	0.9722	0.9597
Average Root Diameter (cm)	0.2410	0.1743
Root Volume (cm^3)	0.9139	0.8104
Number of Root Tips	0.9077	0.8846
Number of Root Forks	0.9091	0.9441
